# Characteristics of street children in Cameroon: A cross-sectional study

**DOI:** 10.4102/phcfm.v8i1.1076

**Published:** 2016-11-18

**Authors:** Samuel N. Cumber, Joyce M. Tsoka-Gwegweni

**Affiliations:** 1 School of Nursing & Public Health, College of Health Sciences, University of Kwazulu-Natal Durban, South Africa

## Abstract

**Introduction:**

The issue of street children is one of the global social problems rising in low- and middle-income countries. These children are vulnerable, but because of a lack of sufficient information, it is very difficult for stakeholders to address their plight in Cameroon.

**Aim:**

To examine the situation and characteristics of street children in three Cameroonian cities.

**Objectives:**

To describe the demographic, socio-economic and behavioural profiles of street children. To identify challenges of street children and to compare the results from the three cities on account of their different settings, cultural history and challenges.

**Materials and methods:**

The study was an analytical cross-sectional survey conducted through researcher-administered questionnaires to 399 street children (homeless for at least a month), in three Cameroonian cities from 1 January 2015 to 30 March 2015.

**Results:**

The majority of the participants were boys, more than 70% were homeless for less than 12 months and poverty was found to be the most common reason for being on the street. Most of the participants earned less than 500CFA francs (USD 0.85), with many of them resorting to begging, drug abuse, sex work and other risky behaviours. Only two of the respondents (0.5%) regarded the public attitude towards them as supportive.

**Conclusion:**

As children roam the streets in search of shelter, food and other basic needs, their future hangs in the balance. Understanding the plight of street children highlights the need for immediate design and implementation of intervention strategies to prevent children from living in the streets and assist those who have become street children.

## Background

Street children have been identified by the United Nations Children’s Fund (UNICEF) as children in difficult circumstances and their rights and welfare remains a growing concern to both national and international bodies.^1^ The existing estimates by UNICEF suggested that there are tens of millions of street-based children, and this number continues to rise in low- and middle-income countries because of increased urbanisation, health challenges of parents, the HIV epidemic, migration and global population growth.^1^

It is reported that street children in every country are developmentally at risk, and the longer they stay on the streets, the worse their situation becomes. This is despite the adoption of the Convention on the Right of the Child by the General Assembly of the United Nations on 20 November 1989.^2^ Evidence shows that while on the streets, these children lack protection, adult supervision and the framework of a family, which is believed to foster healthy development and growth.^2^

The phrase ‘street children’ has not been the only term referring to such children; they have been identified by various terms, including ‘teenage beggars’, ‘street kids’, ‘homeless kids’, ‘street boys’, ‘street bums’, ‘parking boys’, ‘city nuisance’ and ‘children in difficult circumstances’.^3^ They perform informal activities such as begging, wandering, telling lies, stealing and other behaviours necessary for their survival. In most cases, these children do not receive adequate supervision from responsible adults; thus, they are completely left to their own device despite the fact that some still maintain some degree of contact with their families.^4^

Protection, care and upbringing of children remain the duty of parents, and this traditional perspective has existed as an accepted norm of the Cameroonian society for a long time.^5,6,7^ However, this long-established tradition is gradually disappearing because of rapid urbanisation, economic depression and widespread poverty throughout the country, making it difficult for many parents to provide adequate care, love, protection and full attention to their children.^5,6^ Inadequate family income, rural–urban migration, death and other health challenges faced by families in Cameroon contribute to the reasons for some children seeking jobs in the informal economy and thereby directly or indirectly affecting the number of street children in Cameroon.

Because of their less recognised and underdeveloped status, street children are regarded as vulnerable by the Cameroon government, international organisations and other civil societies in the country. However, their well-being and health profile have not been given adequate attention, leaving this vulnerable group of children to survive on the street by themselves, without proper adult supervision.^6^

The aim of the study is to examine the situation and characteristics of street children in three Cameroonian cities. Some specific objectives are to describe the demographic, socio-economic and behavioural profiles of street children; to identify the challenges of street children and to compare the results from the three cities on account of their different settings, cultural history and challenges.

Because of the scarcity of information on street children in Cameroon, it is very difficult for stakeholders to address the plight of these children without knowing their characteristics. It is also important to identify true street children from the general population so as not to waste resources on those who do not fall in the street children category.^1^ Therefore, this study aimed to describe the demographic profile of street children in three urban cities (Bamenda, Douala and Yaoundé) in Cameroon and to answer certain questions from their perspective before proposing any intervention strategies to address their needs. What are their reasons for being on the streets? What challenges do they face while living on the streets? Do they get any type of support?

## Materials and methods

The current study used the phrase ‘street children’ to refer to the population of children who live on the streets in Cameroon, and a street child in this study will be defined as any child, boy or girl, below the age of 18 years, who has taken to the streets (including wastelands, unoccupied dwellings and unfinished buildings) as their habitual abode and source of their livelihood without proper adult supervision.

An analytical cross-sectional survey was conducted during a 3-month period from 1 January 2015 to 30 March 2015, involving 399 street children (320 male and 79 female children), below the age of 18 years in the three urban cities of Cameroon, namely Bamenda, Douala and Yaoundé. With no published prevalence data on street children in Cameroon, a 50% prevalence value of street children in Cameroon was used in calculating the sample size of this study based on the following formula:

$$n=\frac{Z^{2}*P\left(1-P\right)DEFF}{d^{2}}$$ [Eqn 1]

where *n* is the target sample size, *Z* is the *z* statistics for a level of confidence (here *Z* = 1.96, i.e. for a 95% confidence interval), *P* is the estimated proportion of street children. Because there is no documented prevalence of street children in the setting, the calculation assumed 50% (0.5) prevalence (P) because this will produce the largest number of sample size required; *d* is the absolute precision, that is, the width of the confidence interval to be 5% (0.05).

Design effect: Used a clustered-randomised study where the different cities are the primary sampling units. The same number of participants was used in the different cities of the study, giving a design effect to be 1.

Therefore,

$$n=\frac{1-96^{2}\left(0.5\right)\left(1-0.5\right)\left(1\right)}{0.05^{2}}=385$$ [Eqn 2]

It was further assumed that about 5% of the questionnaires would be wrongly filled in or incomplete. Taking these into consideration, 20 more questionnaires (i.e. 0.05*385) were added giving a total of 405 targeted participants. The sample size of 405 street children will represent the street children population as there were no existing data to give an estimate of the total number of street children in any of the chosen urban cities in Cameroon.

The age of street children was self-reported and no identity card was provided; thus, the study relied only on what the street children were reporting orally. Bamenda is the most populated English-speaking city in the country. Douala is a French-speaking city and the economic capital and the most populated city in Cameroon, while Yaoundé, also a French-speaking city, is the capital of the Republic of Cameroon. These three cities were selected because they are known to accommodate large numbers of street children.

We suspected that rapid urbanisation challenges, abject poverty, dysfunctional families and health problems in families among other social problems could account for the increasing number of street children in Cameroon, thus the reason for the study in Cameroon. This study required that participants were primarily school-aged children, 12–17 years old, and who had been on the streets for no less than one month prior to the onset of the study. We felt that the challenges faced by street children who have decided to make the streets their permanent place of residence will be more useful to the study than those who are on the street for few days and back to their homes for few days or weeks.

Recruitment and enrolment took place at the usual gathering points for street children, which were identified as bus stations, train stations, market and city centres and in front of movie houses. With the help of peers, a non-probability technique of snowball sampling was used, whereby each recruited participant led the researcher to other participants.^8^ The study recruited in total 405 street children from the three selected urban cities. We preferred to use snowball sampling to recruit participants because of the highly mobile lifestyle of street children and also because of the limited information on street children in Cameroon. Six participants were excluded because of incomplete information resulting in 399 participants. The local Catholic Church facilities provided private, quiet and neutral locations where the self-designed questionnaires were administered to participants in the different cities. The questionnaires were in both English and French and were also translated into pidgin English, which was a commonly spoken language among street children in the city of Bamenda. The research assistants underwent a 2-day training to ensure all six assistants understood the questionnaire in a similar way before conducting the study. The researcher with the help of six trained research assistants read the questions to participants, as it was presumed that most street children cannot read and if some do they might not understand the questions properly. To protect the identity of the participants, no names were written on the questionnaires, but unique numbers were assigned to each participant for the purpose of the research only. Only the primary researcher had access to the collected data. Though the participants were all minors, obtaining the permission of their parents/guardians in the case of street children was not possible. Only written informed consent form was obtained. The children were first approached by the research team at their meeting point, and the entire study was explained to each one of them including their rights. When a child agreed to participate, he was then given an appointment to meet the team on a certain point (nearest Roman Catholic Church).

The study attempted to not recruit only boys, but included girls even though there were not many who live on the streets and the views of both groups are regarded as important. Moreover, more effort (e.g. like talking to nightclub management to permit us get in contacts with girls under their protection, some street boys took us to specific hideouts where street girls sleep during the day as most work at night) was made to recruit more street girls because they have been shown to be more vulnerable than boys.^5^

After administering the questionnaires, food was given to the participating children and other street children who happened to be around, but no monetary incentive was provided.

The data collected included sex, age, religion, education level, reason for school dropout, time spent on the street, where participants often slept, economic activities, daily average income, how they obtained food and type of contact with families. Statistical analysis of the data included univariate and bivariate calculations of descriptive statistics, such as frequency distributions. Cross-tabulations were used to summarise the data using STATA 13.2.

To qualitatively compare the proportions between the three cities, a chi-square test with Yates correction was used with a *p*-value significance set at 5%.

A pilot study was conducted using a preliminary version of the questionnaire. This was done by testing the questionnaire on a small sample of street children, after which the questionnaire was subsequently modified by adding few questions but mostly modifying those questions which were difficult for the children to understand.

The strength of this study is directed towards the data sample as up to 79 girls were recruited.

Thus far, this is the only study on street children in Cameroon that has been able to recruit so many street girls. Also, the data collection was given special consideration as the participants were minor; thus, the study recruited up to six research assistants to administer the questionnaire to the participants. They were well trained beside their personal experience and achieved academic certificates, which were used as the base for their recruitment.

Nevertheless, the study had some weaknesses, for example, this study was based mainly on self-reported data and the children did not undergo any form of medical or psychological test to get results on their present well-being nor any identification paper were obtained to determine their actual age. Lack of sufficient finance made it difficult to collect data in all 10 regions of Cameroon, which was chiefly the reason only three regions were involved.

## Ethical considerations

The study received ethical approval from the Biomedical Research Ethics Committee (BREC REF: BE331/14), University of KwaZulu-Natal, Durban, South Africa and the Cameroon Bioethics Initiative (CAMBIN REF: CB1/309/ERCC/CAMBIN).

## Results

### Demographic characteristics

Of the total sample of 399 participants, 80.2% were boys and 19.8% girls, with a greater percentage (77.7%) of the participants found in the age group 15–17 years. The ages ranged from 12 to 17 years, with a mean age of 15.4±1.27 years. More than 80% of the participants reported that they were Christians, while less than 10% said that they were either Muslims or traditional/non-religious respectively, with the highest proportion of Muslims coming from Yaoundé. Concerning educational status, 21.3% of participants said they had never been to school, 77.44% had primary education and only 1.3%, mostly from Douala, studied up to secondary school.

The following were cited as reasons for school dropout: family poverty, hatred for school, bullying and maltreatment by teachers. All the demographic variables analysed revealed significant statistical differences between the cities, except for the age groups of the participants (Table 1).

**TABLE 1:** The distribution of demographic characteristics of participants per city (Bamenda, Douala, Yaoundé).

Characteristics	Total	Bamenda (*n* = 125)		Douala (*n* = 137)		Yaoundé (n = 137)		Chi^2^, df
	*N*^o^ (%)	*N*^o^	%	*N*^o^	%	*N*^o^	%	*p*
Sex								
Male	320(80.2)	108	86.4	101	73.7	111	81.0	6.704, 2
Female	79(19.8)	17	13.6	36	26.3	26	18.9	0.035*
Age (years)								
12–14	89(22.3)	29	23.2	31	22.6	29	21.1	0.1682, 2
15–17	310(77.7)	96	76.8	106	77.4	108	78.8	0.919
Religion								
Christianity	331(82.9)	99	79.2	121	88.3	111	81.0	12.21, 4
Islam	36(9.0)	9	7.2	13	9.5	14	10.2	0.016*
Traditional/none	32(8.0)	17	13.6	3	2.2	12	8.8	-
Educational level								
No formal education	85(21.3)	40	32.0	18	13.1	27	19.7	18.71, 4
Primary	309(77.4)	84	67.2	115	83.9	110	80.3	0.001*
Secondary	5(1.3)	1	0.8	4	2.9	0	0.0	-
Reason for school dropout								
No money	212(53.1)	40	32.0	113	82.5	59	43.1	82.06, 4
Did not like school	128(32.1)	56	44.8	11	8.0	61	44.5	< 0.0001*
Bullied in school/teachers not nice	59(14.8)	29	23.2	13	9.5	17	12.4	-

*Source*: Cumber & Tsoka-Gwegweni 2015

* *p*-value is less than 0.05, meaning that the comparison/difference is statistically significant.

### Socio-economic indicators

As shown in Table 2, 44.9% of these teenagers had been homeless for a period of 7–12 months, whereas 35.8% had been homeless for less than 6 months and 19.3% for more than 13 months, with the highest number of the latter group coming from Yaoundé. More than 50% of these children were sleeping in the streets and other places such as markets (19.6%) and old/uncompleted buildings (22.1%) (Table 2). Most of these children had a daily income of less than 500 CFA francs (USD 0.85), with begging being one of their main income-generating activities. A high percentage of street children (88.2%) did not have contact with their families with the highest proportion in Yaoundé (91.9%). From Table 2, the figures show that just under half of the participants (43.6%) had two meals a day, while approximately a fifth of the participants (20.3%) had more than three meals per day.

**TABLE 2:** The distribution of socio-economic indicators of the participants in each city (Bamenda, Douala, Yaoundé)

Characteristics	Total	Bamenda (*n* = 125)		Douala (*n* = 137)		Yaoundé (*n* = 137)		Chi^2^, df
	*N*^o^ (%)	*N*^o^	%	*N*^o^	%	*N*^o^	%	*p*
Time on the street								
< 6 months	143(35.8)	49	39.2	57	41.6	37	27.0	11.93, 4
7–12 months	179(44.9)	59	47.2	57	41.6	63	45.9	0.018*
> 13 months	77(19.3)	17	13.6	23	16.8	37	27.0	-
Where they often sleep?								
On the street	233(58.4)	79	63.2	73	53.3	81	59.1	6.550, 4
Market places	78(19.6)	26	20.8	32	23.4	20	14.6	0.162
Old buildings/shelter homes	88(22.1)	20	16.0	32	23.4	36	26.3	-
Main economic activity								
Begging/support from friends	235(58.9)	73	58.4	83	60.6	79	57.7	8.631, 8
Guarding/washing cars	41(10.3)	13	10.4	15	10.9	13	9.5	0.374
Shoe shinning	23(5.8)	7	5.6	7	5.1	9	6.6	-
Work in small restaurants/bar/clubs	74(18.6)	18	14.4	27	19.7	29	21.2	-
Stealing(pick-pocketing)	26(6.5)	14	11.2	5	3.7	7	5.1	-
Daily average income (in CFA francs)								
0–195	149(37.3)	44	35.2	24	17.5	81	59.1	57.07, 4
200–495	178(44.1)	65	52.0	80	58.4	33	24.1	< 0.0001*
> 500	72(18.1)	16	16.0	33	24.1	23	16.8	-
How they obtain food								
Buying	279(69.9)	84	67.2	108	78.8	87	63.5	4.698, 2
Eating left overs	99(24.8)	41	32.8	29	21.2	29	21.2	0.0955
From drop-in centres	21(5.3)	0	0.0	0	0.0	21	15.3	-
Number or meals per day								
1	144(36.1)	60	48.0	37	27.0	47	34.3	14.68, 4
2	174(43.6)	49	39.2	65	47.6	60	43.8	0.005*
> 3	81(20.3)	16	12.8	35	25.6	30	21.9	-
Number of siblings								
1–4	78(19.6)	43	34.4	22	16.1	13	9.5	28.39, 4
5–8	226(56.6)	53	42.4	87	63.5	86	62.8	< 0.0001*
> 9	87(21.8)	29	23.2	28	20.4	30	21.9	-
Do not know	8(2.0)	0	0.0	0	0.0	8	5.8	-
Frequency of family contact								
No contact	352(88.2)	103	82.4	123	89.8	126	91.9	2.573, 2
Monthly	42(10.5)	17	13.6	14	10.2	11	8.0	0.2762
Other	5(1.3)	5	4.0	0	0.0	0	0.0	-

*Source*: Cumber & Tsoka-Gwegweni 2015

* *p*-value is less than 0.05, meaning that the comparison/difference is statistically significant.

### Reasons for being on the streets

The results show that poverty accounted for 44.4% for participants being homeless. Poverty was self-reported by the participants and was considered as a situation whereby the participants’ parents were unable to provide them with basic needs such as food, clothing, education or medical needs. Authoritarian parents were reported as the second most common reason accounted for (24.1%), the death of a parent (16.8%) and finally dysfunctional family was also self-reported by the children and accounted for (14.9%). Dysfunctional family was defined in this study as a family in which the children experienced one or more of the following feelings: never happy, parents always fighting and quarrelling, one parent or both parents are alcoholic or on drugs, children go without supervision and do whatever they want because parents fail to take up their responsibilities as parents, father always in and out of prison and mother dates other men and not always at home to guard the children.

**FIGURE 1:**
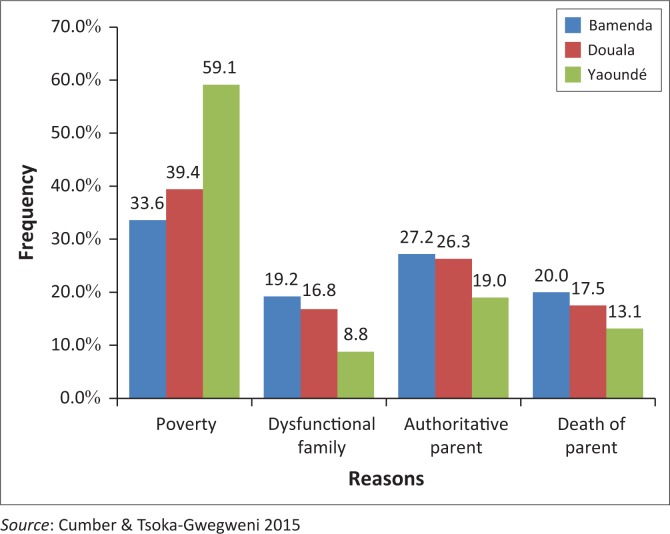
Reasons for being on the street by respondents in each city.

The city of Yaoundé showed the highest percentage of these children citing poverty as the reason for their homelessness (59.1%), followed by Douala (39.4%) and Bamenda (33.6%). The results also showed that authoritative parenting, dysfunctional families and death of a parent are all reasons for homelessness for these teenage children (Figure 1).

### Behaviours and challenges on the streets

From the results, 56.4% of the participants stated that they had been arrested by police in the last 3 months. The reasons for their arrest in descending order are street fighting (18.3%), drunkenness (14.8%), stealing (13.8%) and illegal herb smoking (8.5%). The highest frequency of arrest was found in Douala, followed by Bamenda. Of the total number of participants, 22.31% reported hurting themselves, with the highest percentage coming from Yaoundé. The reasons for this behaviour in descending order are anger (64.0%), drug use (20.2%) and peer pressure (15.7%) (Table 3).

**TABLE 3:** Distribution of some behaviours of respondents in each city (Bamenda, Douala, Yaoundé).

Characteristics	Total	Bamenda (*n* = 125)		Douala (*n* = 137)		Yaoundé (*n* = 137)		Chi^2^, df	
	*N*^o^ (%)	*N*^o^	%	*N*^o^	%	*N*^o^	%	*p*
Police arrest in the last 3 months								
Yes	225(56.4)	59	47.2	112	81.8	54	39.4	56.18, 2
No	174(43.6)	66	52.8	25	18.3	83	60.6	<0.0001*
Reason for being arrested by police								
Never been arrested	178(44.6)	69	55.2	25	18.3	84	61.3	97.87, 6
Stealing	55(13.8)	26	20.8	18	13.1	11	8.0	<0.0001
Street fighting	73(18.3)	11	8.8	52	37.9	10	7.3	-
Was drunk	59(14.8)	13	10.4	34	24.8	12	8.7	-
Illegal herb smoking	34(8.5)	6	4.8	8	5.8	20	14.6	-
Children hurt themselves								
Yes	89(22.3)	31	24.8	20	14.6	38	27.7	7.477, 2
No	310(77.7)	94	75.2	117	85.4	99	72.3	0.024*
Reason for hurting themselves								
Anger	57(64.0)	29	93.6	10	47.6	18	48.7	18.83, 4
Drugs	18(20.2)	0	0.0	7	33.3	11	29.7	0.001*
Peer pressure	14(15.7)	2	6.5	4	19.1	8	21.6	-

*Source*: Cumber & Tsoka-Gwegweni 2015

* *p*-value is less than 0.05, meaning that the comparison/difference is statistically significant.

The results also show that only two participants (0.5%) of the respondents identified the public attitude towards them as supportive, and 73.2% mentioned a lack of attachment and the inability to cope with their life circumstances and life on the streets. More than 80% of the respondents indicated their having fallen sick in the month preceding the study, with identified causes of illness in descending order being mosquito bite (31.1%), exposure to very cold weather (22.8%), drinking dirty water (18.8%) and fatigue from work (10.8%). Up to 11.8% of the respondents accepted self-medication as treatment when ill. More than 50% of the respondents reported having been sexually abused on the street. A large percentage of the participants reported having acquired sexually transmitted diseases (STDs), especially gonorrhoea (53.6%) (Table 4).

**TABLE 4:** Challenges faced by participants in each city (Bamenda, Douala, Yaoundé).

Characteristics	Total	Bamenda (*n* = 125)		Douala (*n* = 137)		Yaoundé (*n* = 137)		Chi^2^, df
	*N*^o^ (%)	*N*^o^	%	*N*^o^	%	*N*^o^	%	*p*
Attitude of public towards street children							
Supportive	2(0.5)	1	0.80	1	0.73	0	0.00	0.023
Hatred	238(59.7)	68	54.4	73	53.3	97	70.8	-
Indifferent	159(39.9)	56	44.80	63	46.0	40	29.2	-
Lack attachment and inability to cope	292(73.2)	84	67.2	98	71.5	110	80.3	0.050
Access to street services	26(6.5)	8	6.4	6	4.4	12	8.8	0.339
Sick in the last month	335(83.9)	113	90.4	107	78.1	115	83.9	0.025
Cause of illness								
Mosquito bite	124(31.1)	43	34.4	40	29.2	41	29.9	11.34, 10
Drinking dirty water	75(18.8)	26	20.8	21	15.3	28	20.4	0.332
Exposure to cold	91(22.8)	27	21.6	32	23.4	32	23.4	-
Fatigue from work	43(10.8)	14	11.2	15	10.9	14	10.2	-
Drug use	6(1.5)	3	2.4	0	0.0	3	2.2	-
Unknown/others	60(15.0)	12	9.6	29	21.2	19	13.9	-
Treatment method sought								
No treatment	194(48.6)	80	64.0	63	45.9	51	37.2	22.31, 4
Self-medication	151(37.8)	33	26.4	62	45.3	56	40.9	0.001*
Traditional doctor	47(11.8)	11	8.8	12	8.8	24	17.5	-
Modern medicine	7(1.8)	1	0.8	0	0.0	6	4.4	-
Type of sexually transmitted disease							
Gonorrhoea	214(53.6)	49	39.2	68	49.6	97	70.8	39.67, 4
Syphilis	29(7.3)	6	4.8	9	6.6	14	10.2	< 0.0001*
Unknown	156(39.1)	70	56.0	60	43.8	26	18.9	-
Sexual abuse on the street								
Yes	257(64.4)	88	70.4	68	49.6	101	73.7	20.19, 2
No	142(35.6)	37	29.6	69	50.4	36	26.3	< 0.0001*

*Source*: Cumber & Tsoka-Gwegweni 2015

* *p*-value is less than 0.05, meaning that the comparison/difference is statistically significant.

**FIGURE 2:**
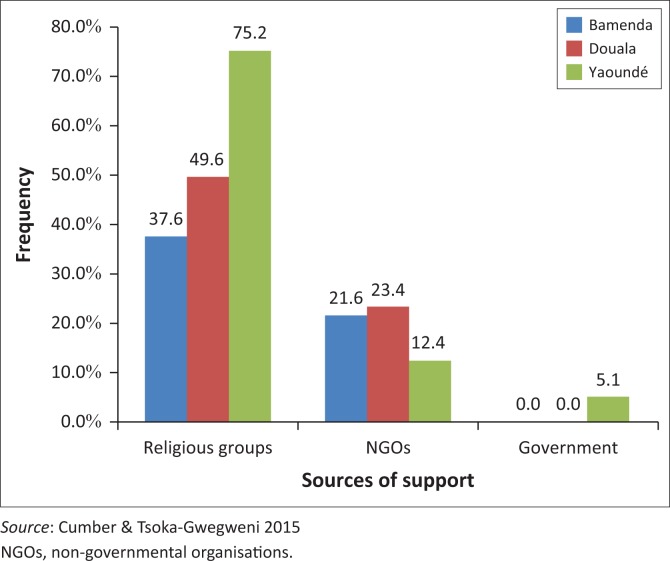
Sources of support for the participants in each city.

Most of the support received by these street children came from religious groups and non-governmental organisations (NGOs), with the Cameroonian government not taking adequate responsibility for this vulnerable population, as confirmed by the very low percentage of respondents who reported having received support from the government (5.1%) and this only in the city of Yaoundé (Figure 2).

## Discussion

The current study focused primarily on the situation of street children in three Cameroonian cities. This population is represented by their demographic profile gathered through self-reports rather than medical examinations. Some studies have focused on self-reporting and/or medical examination of street children which also presented similar results as to those presented in this study on reasons why children run away from home and the challenges they face on the streets in urban cities.^9,10,11,12,13^ Nevertheless, most of the results from this study concur with results reflected in studies that focused on the medical examination and/or other self-reported studies on street children.^9,10,11,12,13^

This study reported several reasons for children to run away from home and onto the streets in the urban city of Bamenda, Douala and Yaoundé in Cameroon. The reasons were self-reported and were listed as follows: poverty, authoritative parents, the death of one or both parents, dysfunctional families among others, with poverty having the highest frequency. The same study also confirmed directly or indirectly that the street also presents opportunities for work for these children, despite the adverse circumstances they have to undergo and endure on the streets as similar results were concurred in other studies conducted in Sudan and Peru.^9,10,11,12,13^

Educational level among street children remains a challenge as majority of the children who participated in this study reported having only primary education and not being able to earn much of an income on the street to provide themselves with basic needs. Most of the children who live on the streets are free, homeless and without any form of adult supervision. Similar results of the majority of street children having only primary education was reported in Tanzania and Nigeria while the majority of those in Sudan had never been to school.^4,9,15^

However, these children on the street do face other challenges; for example, slightly over half of the street children who participated in this study (56.4%) stated that they had been arrested by the police, and the reasons for their arrest were not the same; some were arrested for street fighting, drunkenness, stealing, smoking illegal herbs or for just being a street child on the streets. The highest frequency of reported arrest was in Douala, followed by Bamenda and lastly Yaoundé. This difference in regions might have resulted from the fact that Douala being the commercial capital and Bamenda an English-speaking city have both been controlled by the governor of the region and less represented with high-profile politicians, government authorities and other human right organisations while all these are represented in the Republic’s capital city Yaoundé, thus the reason for the least arrest of street children. In the capital, Yaoundé, where many politicians and the majority of human right activists in the country are based, the police may fear to be exposed as harassing the children, which might account for the low arrest frequency in Yaoundé. Some children reported hurting themselves, with the highest number coming from Yaoundé. The participants did it mostly out of anger 64%, although other reasons such as drug use and peer pressure were also reported.

The various challenges faced by these street children are thus traumatic enough to put their health (physical, emotional, moral and mental) and overall well-being at risk. Other studies reported that the longer children stay on the streets, the more challenges they continue to face on the streets such as poor health, addiction to psychoactive substances, prostitution, getting involved in gangs, mini theft and other criminal activities probably as measures to survive and cope with the challenges; thus, the quality of life and their well-being is at risk, which in turn is more likely to affect their growth and development in the future.^6,11,12,13^

Their adverse circumstances could also be the reasons why some of the street children got involved in risky behaviours, such as abusing psychoactive substances (e.g. alcohol, tobacco and drugs), fighting, commercial sex work, gangsterism and other socially unacceptable behaviours to survive on the streets as reported in some other studies.^5,6^

Also more studies on street children mentioned psychoactive substance use and abuse to be a coping measure and to get courage and confidence for the hardships they face on the streets. Some of the children become addicted because of their duration on the streets. Peer pressure was reported to be a common reason for their initiation.^14,15,16,17^

Their sleeping places (in parks, bus stations, railway stations, in front of shops in the market and other exposed dwellings) render them vulnerable, which is the reason some studies reported why these children join street groups, and other criminal gangs to be able to have an adult companion who can protect them from abuse from the authorities and from other street gangs.^11,12,13,18^

Community support was also reported, but only two of the participants (0.5%) perceived the public attitude towards them as supportive while almost all the street children thought that the public (community) demonstrated hatred, were not friendly, behaved indifferently and held a negative attitude towards them. Because of all the negative perception they have about people in their surrounding or community, it creates a bridge, thus limiting the interaction between them and the public. Thus, when, in facing difficulties (lack of basic needs, shelter, food, sanitation, hygiene, protection and sexual abuse among others), they feel demoralised to run to the same public (including healthcare workers) who already dislike them and call them all sort of names.

The behaviour of street children always has consequences and by grouping with peers to fill the ‘adult gap’ in their lives, their union is more likely to reinforce other harmful habits, such as gambling, smoking, sniffing glue, drug use, pick-pocketing, prostitution and violence.^5,6^ However, the same society that happens to dislike street children still have to deal with the consequences as the society is now exposed to increased crime rates, such as theft, rape, murder, STDs including HIV/AIDS and increased public nuisance in general.^19,21,22^

This study succeeded in recruiting 79 female participants; female perspectives are important and cannot be ignored in order to understand the situation of these children as well as to propose a sustainable intervention for street children in Cameroon. Generally, street girls face more challenges on the street than street boys and they also pose more challenges to social workers and other care workers for street children as most lack the skills and experience to work with street girls as reported in some studies.^11,13^

Support for street children has not been very effective and sustainable, though some participants reported receiving some support from religious groups and NGOs. The above results have been similar to other studies, though there has been a drastic reduction in the support from churches and NGOs. This reduction in support may be because donors have also started channelling their donations to support street children and have diverted their focus towards other issues such as poverty eradication, disaster intervention, migration crisis and other health challenges.^4,11,12,13,22^

## Conclusion

The problem of street children in Cameroon is a visible one that is more likely to affect these young children in the critical stages of their development if immediate attention is not given to their plight. A typical street child in Cameroon is a boy aged 12–17 years, who sleeps on the streets and who has dropped out of school because of lack of money and other support. These vulnerable and exposed children face continuous police arrest, often fall sick and do not have enough money to visit a public hospital or clinic, have no shelter and no food and engage in risky behaviours to survive. Thus, their future hangs in the balance.

## Recommendations

The results show that there are hundreds of street children in the three targeted cities, as the study included 399 children, of whom 79 were girls. These children lack protection because of the absence of any form of adult supervision. They are exposed to violence and all forms of abuse at night. Furthermore, these children need psychological support because of their experiences on the streets. NGOs working with street children should work together with the Cameroon government to provide intensive counselling for these children.

The Cameroon government and all stakeholders working with street children should therefore design specific programmes that will involve these children. Such programmes should include indoors sport and cultural activities, for example, swimming, games, dancing, singing and football competitions, thereby improve the coping measures, self-esteem and the quality of life for these children.

The study recruited 79 girls, which serves as proof of more girls on the streets, as contrary to other studies which recruited fewer street girls. Street children in general are at increased risk of unwanted pregnancy and contracting HIV/AIDS and/or other STDs. Thus, the government, in collaboration with NGOs, should work together in creating a more welcoming health care system that will include and target street children, offering health support, general education and reproductive health for girls.

The results indicated that the education level of street children is poor as over 77.4% dropped out of primary school and 21.3% had no formal education. These children need support to develop skills that they can use to integrate into society in the future. The government and NGOs should develop non-formal education opportunities that can integrate them into the society and also incorporating them into the aforementioned non-formal educational opportunities, with varieties (skills, background, and capabilities) for the government to accommodate different non-educational background so that the street children will have the option of choice so as not to impose a particular education on them.

Two hundred and twenty-five participants (56.4%) have experienced police arrest, as street children are more likely to run foul of the law, but the results indicate that these children lack adult supervision, beg or work under harsh conditions to support their basic needs. Therefore, these children are vulnerable to abuse from police and other authorities, coupled with the fact that the judicial system in Cameroon is underequipped to handle juveniles. They are often confined and tried in open courts along with adults, getting unreasonable sentences without adequate defence. These children are found guilty for being street children. The unequal treatment of street children by the authorities in general is tolerated because they go unpunished even after committing a crime. The government and NGOs fighting for justice and the right of children should work together to see that these children are being given the necessary help rather than prison time. Furthermore, the probationary system should be developed to provide adequate judicial support and other support for street children.

The Ministry of Social and Women Affairs has been ineffective in addressing the issues pertaining to street children, even though it is responsible for developing strategies and conducting research on street children. Intervention strategies exist in theory and laws under the above ministry, but are not being implemented in practice. The ministry of social and women welfare should initiate a process to review existing legislation on strengthening children’s welfare in Cameroon. Furthermore, the government should initiate community mobilisation as a priority in ensuring that duty-bearers and other NGOs working with children are ‘keeping their promises’ to vulnerable children and street children specifically.

Recommendations for further research are important because more studies will identify social and health challenges that need intervention for street children in Cameroon. For example, to best understand the situation of street children, more research is needed, which will focus on their well-being, health, environment and development in order to fill the information gap in Cameroon. The results should be made available to all stakeholders.

There is also a need for research that will focus on medical and psychological examination of street children in Cameroon as this study relied on data from self-reported responses.
